# DNA‐metabarcoding supports trophic flexibility and reveals new prey species for the Galapagos sea lion

**DOI:** 10.1002/ece3.10921

**Published:** 2024-03-01

**Authors:** Diego O. Urquía, Sten Anslan, Pacarina Asadobay, Andrés Moreira‐Mendieta, Miguel Vences, Jaime A. Chaves, Diego Páez‐Rosas

**Affiliations:** ^1^ Maestría en Ecología Tropical y Conservación, Universidad San Francisco de Quito USFQ Quito Ecuador; ^2^ Galapagos Science Center, Universidad San Francisco de Quito USFQ Islas Galápagos Ecuador; ^3^ Institute of Ecology and Earth Sciences University of Tartu Tartu Estonia; ^4^ Deptartment of Biological and Environmental Science University of Jyväskylä Jyväskylä Finland; ^5^ Zoological Institute, Technische Universität Braunschweig Braunschweig Germany; ^6^ Department of Biology San Francisco State University San Francisco California USA; ^7^ Colegio de Ciencias Biológicas y Ambientales Universidad San Francisco de Quito Quito Ecuador; ^8^ Dirección Parque Nacional Galápagos, Unidad Técnica Operativa San Cristóbal Islas Galápagos Ecuador

**Keywords:** bathymetry, DNA‐metabarcoding, ocean productivity, trophic ecology, Zalophus wollebaeki

## Abstract

Tropical ecosystems are challenging for pinnipeds due to fluctuating food availability. According to previous research, the Galapagos sea lion (GSL, *Zalophus wollebaeki*) adopts trophic flexibility to face such conditions. However, this hypothesis comes from studies using traditional methods (hard‐parts analysis of scat and isotopic analysis from tissue). We studied the diet of five rookeries in the southeastern Galapagos bioregion (which harbors the highest GSL density), via DNA‐metabarcoding of scat samples. The DNA‐metabarcoding approach may identify consumed prey with a higher taxonomic resolution than isotopic analysis, while not depending on hard‐parts remaining through digestion. Our study included five different rookeries to look for evidence of trophic flexibility at the bioregional level. We detected 98 prey OTUs (124 scats), mostly assigned to bony‐fish taxa; we identified novel prey items, including a shark, rays, and several deep‐sea fish. Our data supported the trophic flexibility of GSL throughout the studied bioregion since different individuals from the same rookery consumed prey coming from different habitats and trophic levels. Significant diet differentiations were found among rookeries, particularly between Punta Pitt and Santa Fe. Punta Pitt rookery, with a more pronounced bathymetry and lower productivity, was distinguished by a high trophic level and consumption of a high proportion of deep‐sea prey; meanwhile, Santa Fe, located in more productive, shallow waters over the shelf, consumed a high proportion of epipelagic planktivorous fish. Geographic location and heterogeneous bathymetry of El Malecon, Española, and Floreana rookeries would allow the animals therein to access both, epipelagic prey over the shelf, and deep‐sea prey out of the shelf; this would lead to a higher prey richness and diet variability there. These findings provide evidence of GSL adopting a trophic flexibility to tune their diets to different ecological contexts. This strategy would be crucial for this endangered species to overcome the challenges faced in a habitat with fluctuating foraging conditions.

## Graphical abstract

Trophic flexibility is paramount for Galapagos sea lions to thrive in a marginal, tropical habitat. We got solid evidence of trophic flexibility at five different sea lion rookeries and at a broader, bioregional level. This evidence includes the use of prey originating from different habitats and trophic levels by sea lions, a richer diet in more populated rookeries to prevent competition, and the coupling of the sea lion diets with the local bathymetric and oceanographic conditions around each rookery.

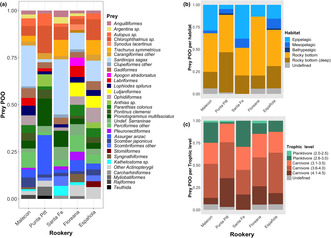

## 1 INTRODUCTION

Top predators are a crucial component of any ecosystem since their ecological conditions make them structuring species (Riofrío‐Lazo et al., 2021; Sergio et al., 2008). Moreover, they function as sentinel species given that their population conditions reflect the functioning of ecosystem at all trophic levels (Drago et al., 2016; Verity et al., 2002). Despite their importance, top predators are intrinsically the least abundant groups in the ecosystems while being the most vulnerable to environmental fluctuations/changes (Hazen et al., 2019; Hutchinson, 1959).

Under fluctuating environmental conditions and/or strong intraspecific competition, some top predators (e.g., pinnipeds) may display trophic flexibility—the ability to take advantage of the most profitable prey under given circumstances—as a strategy to survive (Tyus, 2011; Weise & Harvey, 2008). This ecological adaptation may imply the use of specific prey from different habitats and trophic levels, resulting in some individuals in the population specializing in certain prey to reduce intraspecific competition and increase individual survival (Araújo et al., 2011; Páez‐Rosas et al., 2017). However, these individual preferences are usually flexible so that under changing environmental conditions (e.g., El Niño–Southern Oscillation ENSO), these predators can shift towards other kinds of prey (Páez‐Rosas et al., 2020; Svanbäck & Persson, 2004).

Due to the influence of ocean currents and upwellings, the waters surrounding the Galapagos Islands are often usually productive for a tropical system (Palacios et al., 2006; Schaeffer et al., 2008). This reliance on favorable oceanographic conditions increases the vulnerability of this ecosystem in front of periods when primary productivity low (e.g., ENSO event), which translates into food scarcity across the whole food web (Arnés‐Urgellés et al., 2021; Páez‐Rosas et al., 2020; Salazar & Bustamante, 2003). Such periods are particularly challenging for top predators like the endemic Galapagos sea lion (GSL, *Zalophus wollebaeki*) (Kalberer et al., 2018; Piedrahita et al., 2014), an endangered species whose main threat is starvation due to unfavorable oceanographic conditions (Páez‐Rosas et al., 2021; Riofrío‐Lazo & Páez‐Rosas, 2021).

Trophic flexibility is recognized as a key strategy in GSL for facing the challenges in their habitat with varying prey availability conditions (Blakeway et al., 2021; Páez‐Rosas et al., 2014). The main evidence of trophic flexibility of GSL comes from telemetric, isotopic and diet data, where individuals within the same rookery have been recorded to exploit a high diversity of prey, from different habitats and trophic levels (Páez‐Rosas et al., 2017; Schwarz et al., 2021; Villegas‐Amtmann et al., 2008). Thus, this species can reduce intraspecific competition and obtain an effective dietary response to resources fluctuation (Páez‐Rosas et al., 2017; Schwarz et al., 2022). However, this prey diversity and feeding habitats would not imply that GSL individuals are generalist predators; rather, it demonstrates individual trophic flexibility accompanied by some level of preference for specific prey (Páez‐Rosas & Aurioles‐Gamboa, 2010, 2014).

The bathymetric profile, upwellings, and oceanic current dynamics would be the major oceanographic variables in determining food availability and hence the diet of GSL in different regions of the archipelago (Jeglinski et al., 2015; Wolf et al., 2008). Accordingly, the GSL as a species diversify their foraging strategies to face contrasting oceanographic conditions. For example, animals from the western part of the Galapagos archipelago have affinity to mesopelagic prey from cold, deep waters surrounding the region, Meanwhile, animals from the eastern parts of the archipelago get pelagic and benthic prey from shallower waters over the archipelago shelf (Páez‐Rosas & Aurioles‐Gamboa, 2010, 2014). Despite this, there is still limited information about the trophic behavior of this species since the existing observations come from few rookeries.

The lack of dietary information from several rookeries within the same timeframe precludes the comparison of diets across different ecological contexts. This comparison is necessary to gauge whether and how GSLs adjust their diets to different oceanographical conditions, a key question to actually support trophic flexibility in this species. The comparison of diets among rookeries with different population sizes, and hence different intraspecific competition levels, is also relevant to assess whether trophic flexibility is indeed aiding GSL in preventing competition (Araújo et al., 2011; Bolnick et al., 2003) as previously suggested (Páez‐Rosas & Aurioles‐Gamboa, 2010, 2014). Considering that >60% of the GSL population has perished within the last four decades (Páez‐Rosas et al., 2021; Riofrío‐Lazo & Páez‐Rosas, 2021) mainly due to environmental fluctuations and subsequent food scarcity, to fill such gaps on such a critical aspect as trophic flexibility is paramount for the species conservation.

Here, we describe the diet of five different GSL rookeries in the southeastern Galapagos bioregion by analyzing 124 scats of these animals by a DNA‐metabarcoding approach. The southeastern bioregion is a habitat to more than 60% of the existing GSLs (Páez‐Rosas et al., 2021; Riofrío‐Lazo et al., 2017), yet only a couple of the rookeries of the region have been studied in their trophic ecology (Páez‐Rosas et al., 2017; Páez‐Rosas & Aurioles‐Gamboa, 2010, 2014). Therefore, the applicability of trophic flexibility in other rookeries and at the overall bioregion remains unclear. Previous research about GLS's diet has based on morphological characterizations of hard‐remains in their scats and the analysis of stable isotopes of animal tissue (Páez‐Rosas et al., 2017; Páez‐Rosas & Aurioles‐Gamboa, 2010, 2014).

Since the results from morphological characterizations can be heavily skewed due to differential digestion of remains from different prey (Casper et al., 2007; Peters et al., 2014), and that the results of stable isotope analysis does not provide the specific identity of prey (Deagle et al., 2005; Lerner et al., 2018), it is necessary to implement new techniques to know the feeding patterns of GSL with greater precision. The DNA‐metabarcoding method, detects in theory every prey item egested in a 48 h time‐frame maximum, while not depending on hard‐parts remaining through digestion. This method consists on extracting the total DNA from predator scats to then target an identifiable genetic sequence such as a fragment of the 16S rRNA gene (the DNA barcode). Then, all the barcode sequences from the scat sample are matched to a database to identify the taxa contained therein (de Sousa et al., 2019; Vences et al., 2016). If a complete prey sequence database is available, we may identify all the prey up to the species level (Casper et al., 2007; Nielsen et al., 2018).

Herein, we describe the diet of representative GSL rookeries in the southeastern region through DNA‐metabarcoding. We look for evidence of trophic flexibility—in every studied rookery and then at the bioregional level—by addressing the following questions: (1) Are individuals within the same rookery using prey from different habitats and trophic levels? (2) Are the diets richer in more populated rookeries, potentially as a response to prevent intraspecific competition? (3) How are the different oceanographic contexts at each rookery—namely bathymetry and local productivity patterns—related to the kind of prey and diet diversity observed in GSLs?

## 2 METHODS

### 2.1 Sample collection

**FIGURE 1:**
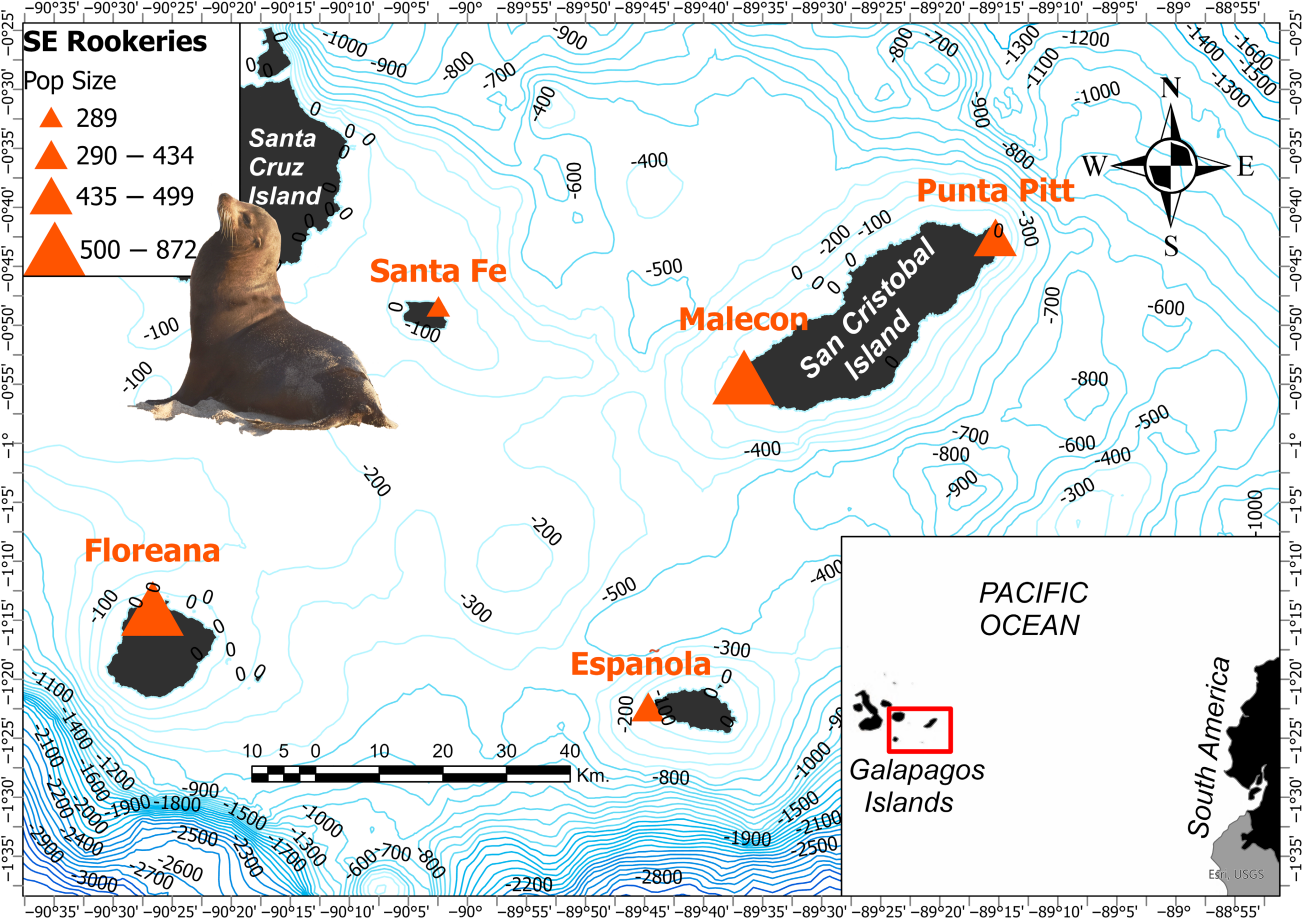
Geographic distribution of studied rookeries in Galapagos. The five localities are shown with a red triangle and bathymetry contour lines (100 m intervals) between islands. The size of the triangle is proportional to population sizes according to Páez‐Rosas et al. (2021).

Our sampling covered the southeastern bioregion of the Galapagos archipelago, from July to August 2021, over the span of 10 days of fieldwork (~2 days/rookery). We sampled five different rookeries, two on San Cristobal Island—El Malecón (0.90072°S; 89.610117°W) and Punta Pitt (0.705069°S; 89.254961°W)—one on Santa Fe (0.8044°S; 90.041073°W), Floreana (1.227624°S; 90.444702°W)—and one on Española (1.369293°S; 89.745053°W) Islands (Figure 1). The highest geographic distance between sampling locations was 145 km (average 50 km).

From the field, we collected around 50 GSL scats (from adults only) per rookery. When possible, scats were immediately collected after the individual defecated. The rest of samples were collected opportunistically, identifying adult individual scats by their size (>10 cm long for solid scats), consistency (solid, or in a high volume of fecal matter when appearing in a liquid consistency), and color (pup and young juvenile scats are always greenish or bright yellowish); we always aimed to collect fresh, still moist, and warm samples only, for guaranteeing good DNA quality. All scats were collected during sunrises and sunsets only, in order to prevent samples being damaged by sunlight. Considering GSLs would spend at the rookery just an average of ~12 h between foraging trips, and that foraging trips may span more than 30 h (Villegas‐Amtmann et al., 2008), we can assume these animals defecated only once per meal in the rookery, avoiding individual pseudo‐replication. Each scat was collected on a clean aluminum foil, removing the surface of it as much as possible; then, the inner part of the scat was mixed for sample homogenization while fresh. The sample was then placed in plastic bags, and immediately stored at −20°C in an electric cooler while on the field and then in our laboratory freezer, until DNA extraction (approx. 3–5 days storage).

### 2.2 Laboratory procedures

From all scats collected in the field, we selected 60 samples from El Malecón rookery, 32 from Punta Pitt and 30 samples from Santa Fe, Floreana, and Española for DNA extraction (total = 182 samples). From the selected samples, ~220 mg wet weight of fecal material was subsampled by cutting several random segments of the frozen scat with a disposable scalpel. All the cut segments per scat were pooled for DNA extraction following the “human DNA analysis protocol” of the QIAGEN QIAamp® Fast DNA Stool Mini Kit. Modifications to the manufacturer's instructions—incorporated following preliminary DNA extraction trials—included the decreasing of incubation temperature from 70 to 50°C, and the final elution of extracted DNA in 100 μL of TAE buffer. DNA concentration was further quantified through spectrophotometry using the Nanodrop 2000®, and integrity was assessed by running the DNA in a 1% agarose gel electrophoresis. In each DNA extraction batch, we included a negative control.

Via PCR, we amplified a ~250 bp region of the mitochondrial large subunit rRNA gene (16S) to target GSL prey items, employing the primers Vert‐16S‐eDNA‐F1: 5′‐AGACGAGAAGACCCYdTGGAGCTT‐3′ and Vert‐16S‐eDNA‐R1: 5′‐GATCCAACATCGAGGTCGTAA‐3′ (Vences et al., 2016). Although these primers were initially designed for targeting vertebrate sequences, we confirmed (in silico) that these possibly amplify also DNA from marine invertebrates (such as cephalopods). This was also confirmed by directly amplifying the expected 16S region from an octopus (*Octopus oculifer*) DNA extracted from a fresh tissue sample, and by obtaining sequencing reads originating from invertebrate parasites. For metabarcoding, the primers were modified by applying a combinational dual‐index framework for multiplexing samples for Illumina sequencing (Vences et al., 2016). A sea lion blocking primer (5′‐TGGAGCTTCAATTAACTTACCCAATCAGAATTTATTC‐3′) was designed to decrease the amplification of GSL DNA in our PCRs. The blocking primer was designed based on the *Zalophus californianus* mitochondrial genome (AM181017; Anderson, 2006). *In silico* comparison of this primer against NCBI database with BLAST (Camacho et al., 2008) revealed no other significant hits than Pinnipeds.

However, we also decided to perform a second round of PCRs without blocking primers, as these may also prevent the amplification of some prey DNA (McInnes et al., 2017); both the PCRs with and without blocking primers were performed in duplicate. For each reaction without blocking primer, we employed 0.2 μL of GoTaq® DNA Polymerase (Thermo Fisher Scientific), 5 μL of reaction buffer, 0.5 μL of dNTP mix, 0.6 μL of each of the Vert‐16S‐eDNA primers, and 2 μL of template DNA (concentration: 10–150 ng/μL), and then we completed 25 μL of reaction mix with dH_2_O. The same reagents and quantities were used in the blocking‐primer assays, except for the addition of 6 μL of blocking primer and the use of 4 μL of template DNA; such amounts were determined after preliminary tests where we succeeded in getting a band at electrophoresis, after testing different concentrations of blocking primer and template DNA. For both assays, the final reaction volume was 25 μL. The thermocycler program included the following steps: (1) Initial denaturation at 94°C for 90 s, (2) denaturation at 94°C for 45 s, (3) annealing at 53°C for 45 s, (4) elongation at 72°C for 90 s, and (5) final elongation at 72°C for 5 min. Steps 2–4 were iterated for 35 times.

All PCR products were loaded on a 1.5% agarose gel for roughly quantifying amplicon concentration, and according to this concentration 1, 2, 4, or 6 μL of PCR product was added to the pooled library (roughly equal library molar concentration at 1–2 ng/μl). The pooled library was gel‐purified using band extraction with the Qiagen MinElute® kit, after which the entire library was concentrated in two columns containing 14 μL of eluate. Library integrity was visualized in a 1.5% agarose‐gel, and the concentration was confirmed in a Qubit 2.0 fluorometer. Library sequencing was conducted on the Illumina MiSeq platform using the MiSeq Reagent Kit v2 for 250 cycles in both directions following the manufacturers' protocol. The resulting raw Illumina sequencing data has been deposited in Sequence Read Archive (SRA) under BioProject ID PRJNA947474.

### 2.3 Bioinformatics and data filtering

Since both, the blocking and no‐blocking primer assays yielded potential prey reads, we pooled the data from both assays and analyzed it together at once. Demultiplexing, reorienting, primer removal, merging, quality, and chimera filtering of Illumina raw reads were done by employing the ‘vsearch OTU workflow’ as implemented in the software package PipeCraft2 v0.1.3 (Anslan et al., 2017), with the following settings: (a) demultiplexing by allowing maximum of 1 mismatch for the index sequences and overlap of 8 bp with cutadapt v3.5 (Martin, 2011); (b) reorient reads to 5′‐3′ as based on primer sequences by allowing 1 mismatch in primers search (reads where primer sequences were not found were discarded at that step) using fqgrep (Indraniel, 2011); (c) cutting primers by allowing 1 mismatch and an overlap of 21 bp using cutadapt v3.5; (d) merging paired‐end reads using VSEARCH v2.18.0 (Rognes et al., 2016) with default settings; (e) quality filtering with VSEARCH by discarding reads with more than maximum error rate (maxee) of 1 and reads containing ambiguous base calls (maxNs = 0); (f) chimera filtering using VSEARCH by pre‐clustering reads using 97% similarity prior *denovo* filtering method. Filtered reads were clustered into operational taxonomic units (OTUs) using a 97% similarity threshold in PipeCraft2‐implemented VSEARCH (‐‐cluster_size, ‐‐iddef = 2). OTUs here are a proxy for prey species; if different OTUs correspond to different species after the taxonomy assignment, they are likely different species indeed. The term OTU has also been used in other trophic ecology studies of pinnipeds (e.g., Berry et al., 2017; Nelms et al., 2019). BLAST v2.11.0+ (blastn; Camacho et al., 2008) was used to assign taxonomy to our OTUs; the reference for taxonomy annotation was the MIDORI 16S database (MIDORI_UNIQ_NUC_GB245_lrRNA_RAW.fasta; Leray et al., 2022), to which we appended the newly obtained 16S sequences for two potential prey species: Galapagos octopus, *Octopus oculifer* (GenBank accession: OQ725638), and mottled scorpionfish, *Pontinus clemensi* (OQ725637).

For mitigating tag‐switching errors (incorrectly assigned reads to a sample; Carlsen et al., 2012), we nullified the occurrence of OTUs with a relative abundance <0.000514 per sample; we determined this threshold based on the maximum relative abundance of sea lion OTUs in our negative control samples. OTUs with no blast hits, OTUs having <70% id% (percentage of identity) against the MIDORI entries, otariid OTUs (i.e., best matches to sea lion‐related taxa), and obvious contaminants (non‐marine taxa) were removed. To remove potential marine environmental contaminants (i.e., “less obvious” contaminants, marine invertebrates mainly) and to correct for cross‐contamination among our samples, we first omitted those OTUs containing equal or less reads than the same OTU at our negative controls. Second, a sample‐specific filtering threshold was set based on the proportion of reads from the obvious contamination sources within every sample; then, we removed all the OTUs in a sample occurring in a proportion under this threshold. Finally, we also omitted OTUs still representing <1% of the sequencing reads in a sample, as well as singletons. These filtering steps also aided in removing species that could be part of the diet of the GSL prey (see Drake et al., 2022, for a full description of filtering methods).

After applying all the filtering steps, we excluded from our records all samples with zero or one prey reads. Then, we also excluded samples in the lowest quartile in terms of prey reads (<37 reads). This resulted in our final prey occurrence matrix of 124 scat samples: 36 from El Malecón rookery, 23 from Punta Pitt, 22 from Santa Fe, 20 from Floreana, and 23 from Española. The taxonomic assignment of the remaining prey OTUs was manually verified to accurately link these OTUs to a given species identification using NCBI blastn server (Madden, 2003) or the lowest possible taxonomic category. Every OTU with an id% ≥96% (see Brassea‐Pérez et al., 2019; Deagle et al., 2013; Thomas et al., 2022) was assigned to the best species‐sequence matching hit, provided the species' presence was confirmed for the Galapagos. Presence‐absence of prey species distributions in the Galapagos was confirmed using the FishBase (Froese & Pauly, 2022), SeaLifeBase (Palomares & Pauly, 2022), and Charles Darwin Foundation Species Checklist (CDF, n.d.) databases. All OTUs with an id% <96% of sequence matching were assigned taxonomic ranking above species level (i.e., genus, subfamily, family, and/or order) using the classification tool, Fast Minimum Evolution tree (NCBI Blastn server: Madden, 2003).

### 2.4 Statistical analyses

We first described the total number of sequencing reads (sequencing coverage) obtained and compared the number of prey reads obtained in the blocking primer experiments vs. the non‐blocking primer experiments through a paired Wilcoxon test. An alpha of 0.05 was considered for all our statistical tests.

Rarefaction curves were plotted for each rockery—employing the *phyloseq* v.1.38 (McMurdie & Holmes, 2013) and *MiscMetabar* v.0.22 (Taudiére, 2022) R packages—to test the power of the applied sequencing coverage in detecting prey OTUs contained in our libraries. Then, all prey data were organized into a presence/absence (0, 1) matrix for subsequent statistical analyses as a conservative and reliable option for avoiding the DNA recovery biases (see Deagle et al., 2019; Nielsen et al., 2018).

#### 2.4.1 Descriptive statistics—Prey richness trends and trophic flexibility

Prey richness—defined simply as the number of different prey items found—(at the rookery and individual level) and rarified richness (assuming a *N* = 20, which is the smallest sample size for any of the studied rookeries) were calculated at the OTU and genus/species level using functions from *phyloseq* and *vegan* v.2.5–7 (Oksanen et al., 2013) R packages. To test whether GSL individuals took more or less prey species in a particular rookery compared to the others, we performed a Kruskal–Wallis rank sum test over the prey richness values per individual across different rookeries. To test for a significant relationship between rarified prey richness (OTU and genus/species levels) and rookery population size, a non‐parametric Spearman correlation was carried out. Population size data were retrieved from Páez‐Rosas et al. (2021); we used population estimations from 2014 since this was a pre‐ENSO year showing normal oceanographic conditions like those of our year of study.

All prey matches across all rookeries were summarized through the percent of occurrence (POO) for each item; the POO is the percentage of the frequency of occurrence of a prey item in a rookery, rescaled so that the sum of the POOs of every prey item in a rookery sum 100% (Deagle et al., 2019). Identified prey were grouped according to five habitat categories: (1) epipelagic (0–200 m deep), (2) mesopelagic (200–1000 m), (3) bathypelagic (1000–3000 m), (4) rocky bottom (0–200 m), and (5) rocky bottom‐deep (>200 m). Prey were also classified according to their trophic level into five categories: (1) Planktivore from trophic levels 2.0–2.5; (2) planktivore, trophic levels 2.6–3.0; (3) carnivore, trophic levels 3.1–3.5; (4) carnivore, trophic levels 3.6–4.0; and (5) carnivore, trophic levels 4.1–4.5. Prey classified into their respective habitat and trophic level categories were summarized through the POO as well. Information about each prey habitat, deepness range, and trophic level were retrieved from FishBase and SeaLifeBase.

#### 2.4.2 Inferential statistics—Diet differences across rookeries

To prevent biases due to samples with low sequencing coverage, we considered only samples with ≥100 prey reads for inferential statistics. We analyzed 33 samples for El Malecón rookery, 18 for Punta Pitt, 20 for Santa Fe, 16 for Floreana, and 18 for Española (total = 105). The inferential statistics described herein examine the differences on the kind of prey and diet variability among rookeries, so we can relate those differences to the distinct oceanographic contexts of each rookery—bathymetry and productivity patterns.

Significant diet composition differences among rookeries were tested via ADONIS (PERMANOVA implemented in the *vegan* R package) for 9999 permutations, based on Jaccard distances among samples grouped in their respective rookeries. The number of reads in every sample was also included as a potential covariate in ADONIS, in order to find out whether the different sequencing coverages (different number of reads) of the different samples is influencing on any dietary variation found. Then, we conducted a pairwise ADONIS (*pairwiseAdonis* v.0.4 package; Martinez‐Arbizu, 2022), employing a Holm correction for *p*‐values (Holm, 1979), to find out which rookeries exactly are driving the diet differences found in ADONIS. The dietary niche overlap among each pair of rookeries was also measured using the Schoener overlap index (Schoener, 1970), which ranges from 0 (meaning no shared prey items) to 1 (full diet overlap); this last analysis was performed with the functions of the R *FSAmisc* v.0.0.3 package (Ogle, 2022). Differences in diet composition among individuals and different rookeries were visualized using the nonmetric multidimensional scaling (NMDS) implemented in the *phyloseq* package in R. NMDS used Jaccard distances among individuals, with a *k* = 3 identified as the number of dimensions needed to faithfully represent diet differences through distances among data points at the NMDS plot (i.e., stress <0.05). The results of the NMDS were plotted through *ggord* v.1.1.6 (Beck, 2022).

For determining whether some rookeries had a broader intra‐rookery diet variation (trophic breadth) than others, we examined the multivariate homogeneity in rookery diet dispersions through Anderson's PERMDISP2 procedure (Anderson, 2006), implemented in the *vegan* R package as well (group centroid; 9999 permutations). A pairwise‐PERMDISP, with a Holm correction for *p*‐values, was used to identify significant dispersion differences between pairs of rookeries. Finally, 95% confidence ellipses for each rookery were drawn in the NMDS to visualize how broad the within‐rookery diet differences were in each case.

Once we had the information about inter and intra‐rookery diet variations, we could associate whether these differences and variation are related with certain bathymetric profiles and productivity patterns. We expected the diet to be different in rookeries with different bathymetries and productivity levels. We also expected more diverse diets in rookeries with heterogeneous bathymetries and higher productivity levels.

Indicator species analysis (ISA) was carried out using *indicspecies* v.1.7.12 (De Cáceres et al., 2022) in R to detect prey items significantly affecting diet composition among rookeries. The species‐site group association function employed for this analysis was the “IndVal” function (9999 permutations). IndVal values closer to 1 are found in the most abundant prey items that are also most exclusively found in a given rookery, or group of rookeries in particular (Dufrêne & Legendre, 1997). ISAs were also performed to test whether prey from a particular habitat or trophic level were more abundant and exclusive for any rookery (or group of rookeries) in particular.

## 3 RESULTS

### 3.1 Metabarcoding data overview and general trends

All collected scat samples containing 16S reads contained also OTUs assigned to sea lion (genus *Zalophus*), confirming that only sea lion scats were collected in the field (99.59% sequence identity with *Zalophus californianus*). Both the blocking and no‐blocking primer assays generated potential prey reads, yet, as expected, the former assay (with blocking primer) contained significantly more prey reads per sample than the latter (Paired Wilcoxon test, *p* = .045).

**FIGURE 2:**
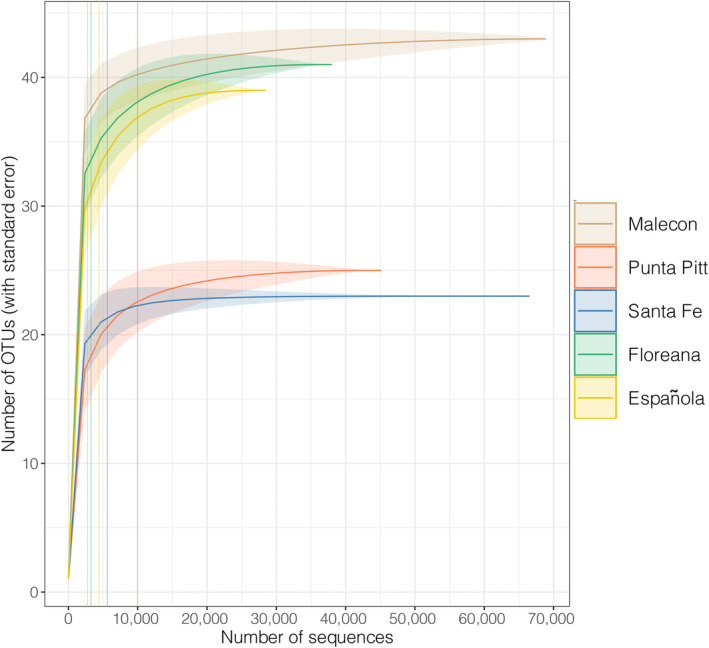
Rarefaction curves of prey OTUs. Each curve corresponds to the number of prey reads sequenced for each studied rookery. Vertical lines represent the number of reads for each rookery corresponding to the coefficient of variation of the rarefaction estimates reaching 0.05.

After filtering our data, a total of 244,189 16S reads were assigned to be potential prey (Data S1). In the retained data, sample with the lowest number of prey reads contained 39 reads, while the one with the highest had 31,731 reads (mean per sample = 1969 reads). Rarefaction curves for all five rookeries (pooled samples per rookery) successfully reached an asymptote, at ~9000 reads on average. The most reads were obtained from El Malecón and Santa Fe rookeries, and the least reads from Floreana and Española (Figure 2).

The vast majority (99.80%) of prey reads belonged to bony ray‐finned fish (Actinopterygii), while the remaining reads (0.20%) belonged to sharks, rays, and a squid. Data filtering provided a total of 98 prey OTUs (Data S2), of which 58 could be identified up to the species level, 30 to the genus level, 5 to family, and 5 to order only (Data S1). All blast hits used for taxonomic assignment had an e‐value <4.89E−41. The prey OTUs found were classified into 49 unique species (46 Actinopterygii, 2 Chondrichthyes, 1 squid), 70 genera (66 Actinopterygii, 3 Chondrichthyes, 1 squid), 48 families (44 Actinopterygii, 3 Chondrichthyes, 1 squid), and 31 orders (27 Actinopterygii, 3 Chondrichthyes, 1 squid). The 98 prey OTUs were classified into 85 unique taxonomic entities, either species, genus, family, or order. A total of 22 of these OTUs (~22%) were matched with an id% ≥96% with species that are not reported in the Galapagos Islands.

The Pacific sardine (*Sardinops sagax*) produced the highest number of prey reads (126,408 reads; 51.76% of the total prey reads obtained), while being the most frequent prey in this study (found in 52 of the 124 scat samples, equivalent to the 42% of total samples). Other recurrent prey in terms of reads and frequency included the greeneyes (*Chlorophthalmus* sp.; 31,700 or 12.98% of the reads; 22% of scat samples), razorback scabbardfish (*Assurger anzac*; 24,037 reads or 9.84%; 19% of scats), Pacific creolefish (*Paranthias colonus*; 7842 reads or 3.21%; 8.9% of scats), and the threadfin bass (*Pronotogrammus multifasciatus*; 4497 reads or 1.8%; 18% of scats) (Data S1).

### 3.2 Prey richness and trophic flexibility

**FIGURE 3:**
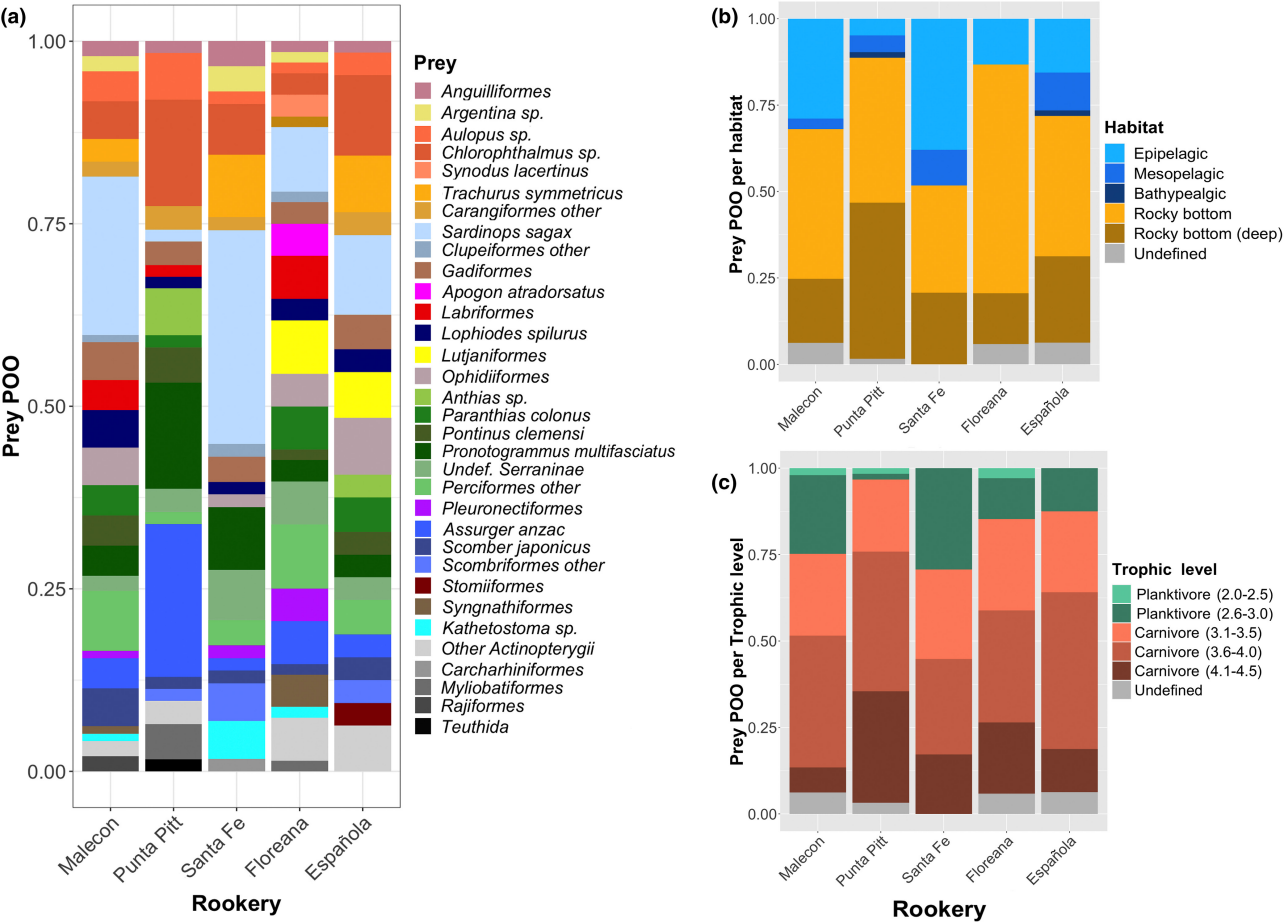
Bar Plots of the relative abundance of preys per rookery based on the ‘Percent Of Occurrence’ (POO). (a) Prey abundance with POO >5% in at least one rookery correspond to colored boxes showing species/genus, POO between 1 and 5% correspond to boxes classified by Order, POO <1% to Class. (b) Relative prey abundance according to habitat: Epipelagic (0–200 m), mesopelagic (200–1000 m), bathypelagic (1000–3000 m), rocky bottom (0–200 m), and rocky bottom‐deep (>200 m). (c) Relative prey abundance classified according to the trophic level.

Prey came from different habitats and trophic levels at all the rookeries. In particular, prey originating from the epipelagic, rocky bottom and rocky bottom‐deep habitats were recurrently detected in all the five rookeries (at different proportions, though) (Figure 3b). Likewise, all the rookeries showed prey from at least four different trophic levels, spanning from high level planktivores (trophic level = 2.6–3.0) to high level carnivores (trophic level = 4.1–4.5). Carnivore prey showed in general higher POOs than planktivore prey (Figure 3c). Even if some individuals showed >10 different prey consumed, in average few prey OTUs (Table 1) and species/genera (Table 2) were detected per individual sample across all the five rookeries equally (OTUs: Kruskal‐Wallis test, $\chi_{4}^{2}$ = 2.28, *p* = .683; species/genera: $\chi_{4}^{2}$ = 3.13, *p* = .537).

**TABLE 1:** Prey richness statistics per rookery at the OTU level. The population size (measured in 2014) for each rookery is shown in column “Pop. Size”.

Rookery	*N*	Prey richness	Prey richness w/rarefaction^a^ (95% CI)	Average prey richness/individual [med.] (range)	Pop. Size 2014^b^
Malecón	36	43	30 (29.2–30.8)	2.69 [2] (1–12)	872
Punta Pitt	23	25	23 (22.5–23.5)	2.70 [2] (1–6)	499
Santa Fe	22	23	22 (21.6–22.4)	2.64 [2] (1–7)	289
Floreana	20	41	41	3.40 [2] (1–11)	731
Española	23	39	36 (35.5–36.5)	2.78 [2] (1–8)	434

*Note*: Prey richness here is defined as the number of different prey OTUs found.

^a^ Rarefaction standardized for *N* = 20.

^b^ Population size data from Páez‐Rosas et al. (2021).

**TABLE 2:** Prey richness statistics per rookery at the species/genus level.

Rookery	*N*	Prey richness	Prey richness w/rarefaction^a^ (95% CI)	Average prey richness/individual [med.] (range)	Pop. Size 2014^b^
Malecón	36	33	24 (23.4–24.6)	2.42 [2] (1–10)	872
Punta Pitt	23	22	20 (19.5–20.5)	2.61 [2] (1–6)	499
Santa Fe	22	21	20 (19.6–20.4)	2.45 [2] (1–6)	289
Floreana	20	36	36	3.00 [2] (1–8)	731
Española	23	31	29 (28.5–29.5)	2.43 [2] (1–7)	434

*Note*: The population size (2014) for each rookery is shown as well. Prey richness here is defined as the number of different prey species/genera found.

^a^ Rarefaction standardized for *N* = 20.

^b^ Population size data from Páez‐Rosas et al., 2021.

Floreana, Española, and El Malecón were identified as the sites with the highest prey richness consumed; meanwhile, Santa Fe and Punta Pitt displayed the lowest prey richness (Tables 1 and 2, Figure 2). No significant relationship was detected between rookery population size (estimation for 2014) and prey richness, both in terms of OTUs (Spearman correlation, *p* = .450, rho = 0.50; Table 1) and species/genus (*p* = .493, rho = 0.41; Table 2).

### 3.3 Diet variation among rookeries

**FIGURE 4:**
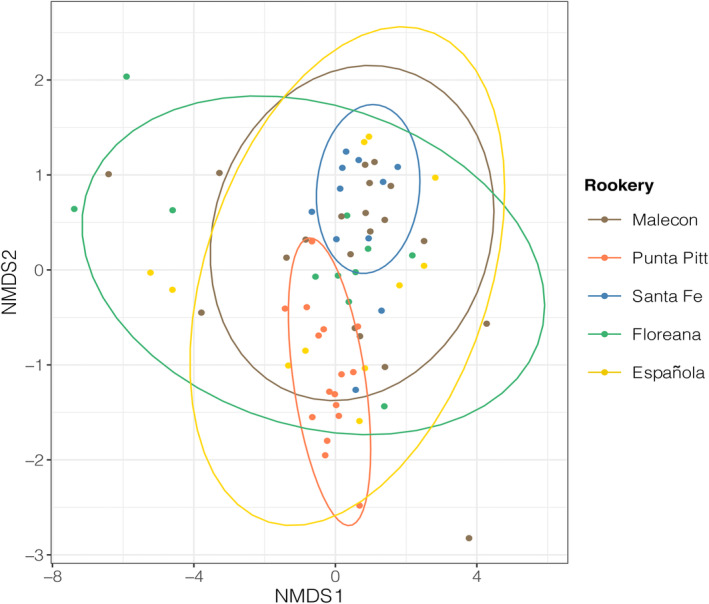
Nonmetric multidimensional scaling (NMDS) plot with 95% confidence ellipses showing differences and overlaps in diet composition among GSL individuals from different rookeries (Jaccard distances; *k* = 3). The distances plotted among data points reflect well the actual diet differences (stress = 0.048).

Diet composition varied significantly among rookeries as tested via ADONIS (*Model*‐*F*_4,99_ = 3.150, *p* < .001, *R* ^2^ = .112) and visualized in the NMDS (Figure 4; stress = 0.048). This difference was mainly driven by the diet composition in Punta Pitt differing from all other rookeries, especially from Santa Fe; however, the diet in Santa Fe also differed from the one in Floreana and Española (Table 3; Figure 4). The number of reads per sample had no significant effect on the observed diet variation (ADONIS, *Model*‐*F*_1,99_ = 1.118, *p* = .307, *R* ^2^ = .010). As measured by the Schoener index, Punta Pitt also showed the lowest dietary overlap with other rookeries, especially with Santa Fe (Table 4).

**TABLE 3:** Pairwise ADONIS and PERMDISP TukeyHSD results for examining, for each pair of rookeries, the significance of differences in diet composition and of multivariate homogeneity in diet dispersions, respectively.

	Malecón	Punta Pitt	Santa Fe	Floreana	Española
Malecón	–	0.492	0.137	0.492	0.375
Punta Pitt	**0.001**	–	0.365	**0.047**	**0.014**
	*0.131*				
Santa Fe	0.496	**0.001**	–	**0.009**	**0.002**
	*0.027*	*0.219*			
Floreana	0.691	**0.002**	**0.028**	–	0.518
	*0.022*	*0.086*	*0.072*		
Española	0.671	**0.002**	**0.039**	0.691	–
	*0.024*	*0.088*	*0.062*	*0.025*	

*Note*: The ADONIS results are under the diagonal (*p*‐values are up, *R* ^2^ values are down in italics); the *p*‐values for the PERMDISP TukeyHSD are above the diagonal. The *p*‐values shown are corrected through Holm's method. Values in bold correspond to significant differences.

**TABLE 4:** Schoener's overlap index for each pair of rookeries depicting dietary niche overlap (0 = no prey items shared; 1 = full niche overlap).

	Malecón	Punta Pitt	Santa Fe	Floreana
Punta Pitt	0.255	*–*	*–*	*–*
Santa Fe	0.502	0.227	*–*	*–*
Floreana	0.493	0.280	0.391	*–*
Espanola	0.487	0.335	0.391	0.407

While 11.18% (ADONIS, SS_A_ = 4.814) of diet variation was explained by the rookery grouping variable, a majority of 87.83% (SS_W_ = 37.820) was explained by an important within‐group variation. When visualizing probability ellipses at the NMDS, Floreana, Española, and El Malecón — in that order — displayed the highest within‐rookery diet variations, while Punta Pitt and Santa Fe showed the most restricted diets (Figure 4a). The diet multivariate dispersions of the different rookeries were not homogenous (PERMDISP, *F*_4,100_ = 4.481, *p* = .003); in particular, the diet in Santa Fe and Punta Pitt had a significantly different (smaller) dispersion compared to that seen in Floreana and Española (Table 3).

The high frequency of *A. anzac* and the absence of *S. sagax* in the diet of Punta Pitt were the major drivers of this rookery's diet differentiation when compared to other rookeries. Prey from the genus *Anthias* had also an important role in differentiating the diets from Punta Pitt and Española from the remaining rookeries. Meanwhile, *Fistularia commersonii* contributed to differentiate the diet in Floreana (ISA: Table 5).

**TABLE 5:** Prey OTUs driving the diet differentiation for each rookery or group rookeries according to the indicator species analysis (ISA).

Rookery/ies	Assig. Taxonomic rank	IndVal	*p*
Punta Pitt	*Assurger anzac* (OTU_134)	0.590	<.001
Floreana	*Fistularia commersonii* (OTU_152)	0.354	.025
Punta Pitt and Española	*Anthias* sp. (OTU_118)	0.373	.023
All except. Punta Pitt	*Sardinops sagax* (OTU_033)	0.743	<.001

*Note*: The IndVal statistic (~1 = prey highly abundant and exclusive for one rookery or group of rookeries) and its significance *p*‐value are shown as well.

When grouping the prey according to their habitat, we found that epipelagics were significantly associated to all rookeries excepting Punta Pitt (ISA, IndVal = 0.788, *p* < .001). Mesopelagic prey were also a significant driver differentiating the diets of Santa Fe and Española from the other three rookeries (IndVal = 0.461, *p* = .018). Rocky bottom‐deep prey tended to have a higher POO in Punta Pitt than in the other rookeries; similarly, the few bathypelagic prey items detected, were exclusive to Punta Pitt and Española (Figure 3b). When prey were grouped into trophic levels, planktivorous prey from trophic levels 2.6–3.0 were significantly associated to all rookeries excepting Punta Pitt (ISA, IndVal = 0.766, *p* < .001), where in fact these prey were almost absent (Figure 3c). Meanwhile, carnivores from trophic levels 4.1–4.5 were significantly associated to all rookeries excepting El Malecón (IndVal = 0.671, *p* = .001).

## 4 DISCUSSION

### 4.1 GSL prey revealed by DNA‐metabarcoding

In agreement with previous research based on hard remains from scats (e.g., Dellinger & Trillmich, 1999; Páez‐Rosas et al., 2017, 2020; Páez‐Rosas & Aurioles‐Gamboa, 2010, 2014), bony‐fish contributed to the vast majority of the GSL diet as in our study. Moreover, 48 of the OTUs we found (56.5%) corresponded to newly reported prey. These included low‐frequency prey mainly, but also some frequent prey such as *A. anzac*, the most common in the Punta Pitt rookery. The aulopiform *Chlorophthalmus* sp. was also a frequent prey reported for the first time in the southeastern bioregion, reported before only from western rookeries (Dellinger & Trillmich, 1999). Most of the newly reported prey in our study—including *A. anzac* and *Chlorophthalmus* sp.—are deep‐sea fish from bathypelagic and rocky bottom‐deep habitats (>200 m).

Our study is the first in reporting Chondrichthyes in the GSL diet. Since sharks and rays lack otoliths (Fowler et al., 2005), and their denticles are apparently undetectable in GSL scats, these species might have eluded their detection in previous GSL diet studies based on the analysis of hard remains (Dellinger & Trillmich, 1999; Páez‐Rosas et al., 2017, 2020; Páez‐Rosas & Aurioles‐Gamboa, 2010, 2014). Likewise, several of the undetected fish prey in other studies could have otoliths that either digest easily or are not recorded in existing otolith guides (Casper et al., 2007), this being especially feasible for understudied deep‐sea species.

Other factors besides the method could also explain why we found so many new prey for the sea lion. For example, the inclusion of the Punta Pitt rookery—whose diet has never been studied—lead us to unveil several novel deep‐sea prey species that were found in this particular rookery; the diet items of other rookeries as Española and Santa Fe had not been previously studied deeply as well, excepting by a study that identified prey to the family level only (Salazar & Bustamante, 2003).

Diet variability over time could also explain why we found novel prey, as well as why we did not find many prey reported previously (around 42 taxa, including some frequent prey). In the widely studied El Malecón rookery, *Chlorophthalmus* sp. was not reported as a GSL prey before, while *S. japonicus* was reported as a minor prey only (Páez‐Rosas et al., 2017, 2020; Páez‐Rosas & Aurioles‐Gamboa, 2010, 2014); note these two were frequent prey in our study. Additionally, prey reported frequently in previous years in El Malecón such as *Opisthonema berlangai* (Páez‐Rosas et al., 2017, 2020; Páez‐Rosas & Aurioles‐Gamboa, 2010, 2014). were absent in our records. Such temporal diet shifts in GSL would be linked with environmental changes, like the Pacific decadal oscillation, or the strong ENSO event in 2015–2016, together with the trophic flexibility GSLs must have to face those changes (Páez‐Rosas et al., 2020; Villegas‐Amtmann et al., 2013). Similar temporary changes have been observed in the diet of the California sea lion in the Northeast Pacific; this population went from being a *S. sagax* specialist in 1998, to having a fairly varied diet by 2016 where *S. sagax* was a minor prey item, this presumably in response to climatic variations in the region (Robinson et al., 2018; Weise & Harvey, 2008).

### 4.2 Lessons and limitations from the DNA‐metabarcoding

The DNA‐metabarcoding method does not rely on the integrity and identification of otoliths or any other hard remains in scats (Nielsen et al., 2018). Thereby, the implementation of this methodology in the GSL trophic ecology, as in other pinnipeds (e.g., Berry et al., 2017; Brassea‐Pérez et al., 2019; Jeanniard‐du‐Dot et al., 2017; McCosker et al., 2020; Thomas et al., 2022), has unveiled not only tens of novel bony‐fish prey, but sharks, rays, and soft‐bodied animals such as mollusks. Despite this, several limitations arose in the implementation of this technique in a short time span.

Prey quantification via DNA‐metabarcoding (i.e., the translation of the number of sequencing reads into prey biomass) still remains as a speculative exercise in field studies (Nielsen et al., 2018; Thomas et al., 2014). However, the inclusion of several samples per rookery in combination with values from the POO could overcome this limitation by providing a reliable contribution of each prey item to the total GSL diet (Deagle et al., 2019). Thus, we acknowledged the importance of prey such as *S. sagax*, *Chlorophthalmus* sp., *A. anzac*, *P. colonus*, and *P. multifasciatus* for the southeastern GSLs (Data S1) at least during the study period time frame.

Issues regarding sequencing coverage and species detection could also have hindered our ability to provide a complete diet composition. Despite the fact that there were fewer sequencing reads than we expected, our rarefaction curves showed that our approach could detect every prey OTU contained in our sequencing libraries. Nonetheless, we still suggest using higher concentrations of blocking primer when employing it to recover more prey sequences while decreasing the undesired yield of predator reads. If there is a concern about the possibility of amplifying prey due to blocking primers mismatches (see McInnes et al., 2017; Piñol et al., 2015), a separate assay without blocking primers could be carried out for testing this and to capture additional prey that perhaps are being missed in the blocking primer assay.

Another limitation we faced was that some prey OTUs could not be taxonomically identified to the species or genus level. Moreover, 22 of the OTUs were matched to species that would not be present in the Galapagos Islands. Such unmatched OTUs most likely represent missing sequences in the sequence reference database, highlighting the need to increase global DNA barcode reporting efforts(Adamowicz, 2015; Leray et al., 2022) and particularly, in the Galapagos (Chaves et al., 2023).

### 4.3 Trophic flexibility in GSL

Our results contribute to the body of research that indicates trophic flexibility as a recurrent behavior in GSL. Across all the studied rookeries, we found prey coming from diverse trophic levels and habitats, just as previous research suggesting trophic flexibility in this species did (Páez‐Rosas et al., 2017; Páez‐Rosas & Aurioles‐Gamboa, 2010). Our results are also concordant with the three foraging strategies described before for the GSL: epipelagic, mesopelagic and benthic (Schwarz et al., 2022; Villegas‐Amtmann et al., 2008). The use of different feeding habitats is a usual characteristic of high trophic level predators that face high intra‐specific competition, but low inter‐specific competition (Kernaléguen et al., 2015). This is exactly the case of GSL in the southeastern bioregion, where this species presents the highest abundance while lacking a potential competitor, the Galapagos fur seal (*Arctocephalus galapagoensis*), present only in the northern and western bioregions of the archipelago (Páez‐Rosas et al., 2021; Riofrío‐Lazo & Páez‐Rosas, 2021).

Specialization at the individual level in GSL (Páez‐Rosas et al., 2017; Páez‐Rosas & Aurioles‐Gamboa, 2010) could also be partially supported by our finding of few prey items in most individuals (2–3 OTUs per individual in average), plus intraspecific variation in the diet of each rookery. Nevertheless, given that our DNA‐metabarcoding data would only reflect dietary information from the last foraging trip of an individual, (<5 h, which is the duration of the passage of digesta in California sea lions; Helm, 1984), dietary seasonal adjustments and time‐stable individual specialization are missing in support here. In any case, our results supplement diet, telemetry, and stable isotope studies, where flexible and dynamic individual specialization is reported for GSL at the level of days (Páez‐Rosas & Aurioles‐Gamboa, 2010; Villegas‐Amtmann et al., 2008), months (Páez‐Rosas et al., 2017; Urquía & Páez‐Rosas, 2019) and even years (Drago et al., 2016).

Prey richness and rookery population size were not significantly correlated in our study. However, note El Malecón and Floreana rookeries—the most populated in the southeastern bioregion and in the whole archipelago (Páez‐Rosas et al., 2021; Riofrío‐Lazo et al., 2017)—displayed the highest prey richness. Meanwhile, the less populated rookeries—Punta Pitt and Santa Fe—had a lower prey richness. Although not statistically (perhaps due to the small sample size), both observations support trophic flexibility in GSL, as a strategy resulting from population density (Páez‐Rosas et al., 2017), with more diverse diets in more populated rookeries where intraspecific competition would be stronger (Araújo et al., 2011; Bolnick et al., 2003). Similar trends have been reported in the California sea lion, *Zalophus californianus*, generalizing this behavior across pinnipeds (Porras‐Peters et al., 2008; Rosas‐Hernández et al., 2019). However, the fact that Española had a richer diet despite being less populated than Punta Pitt, and the lack of a significant correlation between prey richness and population size overall, could suggest an influence of other factors such as the different oceanographic contexts (e.g., bathymetry and productivity patterns) of each rookery (see Section 4.4).

The exploitation of different prey sources aids pinnipeds such as GSL to inhabit in tropical environments (Páez‐Rosas et al., 2017). Trophic flexibility has also been recognized as crucial for other extant tropical pinnipeds such as the Hawaiian monk seal (*Neomonachus schauinslandi*) (Kienle et al., 2019) and the Galapagos fur seal (Páez‐Rosas et al., 2012: Riofrío‐Lazo & Páez‐Rosas, 2021). Some populations of the Californian sea lion are also distributed in the tropics and subtropics; these have been regarded as plastic specialists, just as GSL. Due to its low and fluctuating marine productivity, tropics are marginal habitats for pinnipeds in general (Costa et al., 2006). Here, these species rely on the dynamics of oceanic currents and upwellings, which could eventually lead to extended and frequent starvation periods as seen during ENSO cycles in the Tropical Pacific (Capotondi et al., 2015; Soto et al., 2004). Therefore, our results confirm that diet plasticity is a paramount strategy for GSL to thrive in a challenging environment, not only in a few populations as already demonstrated (Páez‐Rosas & Aurioles‐Gamboa, 2010), but overall throughout the southeastern bioregion where most of the species is distributed (Páez‐Rosas et al., 2021).

### 4.4 Oceanographic conditions and diet differentiation among rookeries

Before assessing how the different oceanographic conditions at each rookery are related to the GSL diet, we firstly had to make sure these animals are only consuming prey in the area around their respective rookery. This premise is supported by the site fidelity shown in both, female and male GSL, for foraging and breeding activities (Drago et al., 2016; Kalberer et al., 2018; Meise et al., 2013; Piedrahita et al., 2014). The distance female GSL travel in their foraging trips is in average of 27–46 km from the rookery (Jeglinski et al., 2015; Páez‐Rosas et al., 2017; Villegas‐Amtmann et al., 2008), and can dive up to 600 m deep (Riofrío‐Lazo & Páez‐Rosas, 2021). Thus, our dietary estimations should trustfully capture the local effects dictated by the bathymetry and the oceanographic dynamics around each rookery.

We also ensured other confounding variables (i.e., temporality, sex, and age category) did not influence in the diet differences found across rookeries, so that we just keep the desired effects of local oceanographic conditions. For example, Drago et al. (2016), by analyzing stable isotopes, showed the lack of long‐term feeding patterns differences between sexes in GSL. Even if prey species differ between sexes, we still don't expect this to mark dietary differences among rookeries, since all our study sites are pupping rookeries where females are always predominant in the same ratio (55.5% females and 13.5% males in the rookeries; Páez‐Rosas & Aurioles‐Gamboa, 2010, 2014); thus, in all rookeries the probability of sampling male scats was low; hence, sex should not be an important explanatory variable for the diet variation we found among rookeries. Samples were collected from all rookeries during the same season to control for seasonal or breeding‐related diet variations. Similarly, we showed that the different sequencing coverages (different number of reads) of the different samples did not influence the dietary variation found. Finally, we sampled adult‐sized scats only to control for individual size and age effects. However, differences have been found in the diving abilities and foraging habitats of adults and juveniles (Jeglinski et al., 2015); therefore, there could be dietary differences between adults and subadults of different ages as well, that may be also adding some variability in our dietary results besides oceanographic conditions; hence, some caution should be taken in this regard.

The most significant diet differentiations were found between Santa Fe and Punta Pitt, both rookeries with opposite bathymetric profiles. Santa Fe Island is located in the central part of the insular shelf surrounded by shallow waters (e.g., 200 m isobath.), whereas Punta Pitt, located on the eastern extreme of San Cristóbal island, is characterized by a pronounced bathymetry with isobaths exceeding depths of 1000 m (Páez‐Rosas et al., 2017). This may explain why the diet in Punta Pitt is characterized by bottom fish, several of which being deep‐sea species including *A. anzac*, *Anthias* sp., and *Chlorophthalmus* sp. (Froese & Pauly, 2022). These fish were either absent or in a low POO in Santa Fe reported species.

Marine productivity differences between Punta Pitt and Santa Fe may also explain the non‐concurrent diets of these sites. The Cromwell current upwelling is the main supply of nutrient‐rich, cool water in the Galapagos archipelago (Palacios et al., 2006; Schaeffer et al., 2008). The incidence of this current is the strongest in the western Galapagos bioregion, but its effects also extend eastwards (Palacios et al., 2006) generating a west–east productivity gradient (Palacios, 2004). In consequence, due to its westernmost location, the productivity in Santa Fe is higher than in Punta Pitt (Palacios, 2002; Schaeffer et al., 2008). This leads to a higher phytoplankton abundance in Santa Fe, which in turn is attractive for epipelagic planktivorous fish such as *S. sagax* (Froese & Pauly, 2022), the most frequent prey in this rookery according to our study. In Punta Pitt, where primary productivity is rather low, *S. sagax* and other epipelagic fish relying on plankton are practically absent in the GSL diet. Instead, most of the prey here were high‐trophic level carnivore fish from the sea bottom, which are less impacted by changes in surface productivity (Ñiquen & Bouchon, 2004).

El Malecón, Floreana, and Española showed a higher prey richness and within‐rookery diet variability than Punta Pitt and Santa Fe. This might be related to the geographical location of those three rookeries, with close access to both, epipelagic prey in the shallow shelf (as seen in Santa Fe) and deep‐sea prey out of the shelf (as seen in Punta Pitt). In any case, these diets showed more overlap with Santa Fe (especially due to consumption of *S. sagax*) than with Punta Pitt. This closer relationship with the diet recorded in Santa Fe could result from a higher energy gain from consuming epipelagic prey (epipelagic fish have a higher lipid content, and a lower energetic investment is required for accessing them; Drago et al., 2010), that make up large schools during productive years (Schwarz et al., 2022). However, not every animal from the three rookeries consumed a pelagic diet; some individuals within each rookery showed rather preferences towards prey from the sea bottom. These results agree with research at El Malecón rookery, where a group of female GSL fed on epipelagic prey over the continental shelf, while another group fed on carnivore fish from deeper waters, off the shelf (Páez‐Rosas et al., 2017; Páez‐Rosas & Aurioles‐Gamboa, 2010). This alimentary niche partitioning would rely on the individuals' body size and diving performance (Villegas‐Amtmann et al., 2008); moreover, in the case of females, this would also depend on whether they are pregnant and on their pups' age (Urquía & Páez‐Rosas, 2019; Villegas‐Amtmann et al., 2017).

The effects of marine currents may also explain the broader diets found in Floreana, Española, and El Malecón rookeries. Floreana and Española are highly influenced by a southern “branch” of the Cromwell current upwelling that extends towards the east, as well as by the cold Humboldt Current coming from the south (Schaeffer et al., 2008; Tompkins & Wolff, 2016). El Malecón rookery, located in the southwestern tip of San Cristobal Island, also receives these influences, contrary to Punta Pitt, located on the same island but in the northeastern side (Palacios, 2004). These currents' influences would increase productivity, environmental heterogeneity, and hence potential prey diversity around Floreana, Española, and western San Cristóbal (Edgar et al., 2004; Moity et al., 2019), contributing thereby to the broader diets found there.

Our results showed GSL also adjust its diet according to the oceanographic conditions (i.e., bathymetry and productivity) of their respective rookeries and foraging grounds. The ability of GSL to mold its foraging behavior according to the ecological context is crucial to face environmental change (Páez‐Rosas et al., 2020; Tyus, 2011), and to prevent competition for similar resources among individuals from different rookeries but with overlapping foraging ranges (Jeglinski et al., 2015; Páez‐Rosas & Aurioles‐Gamboa, 2014). This trophic flexibility in GSL could be ratified thanks to our simultaneous examination of five different rookeries within the same bioregion yet under different oceanographic contexts; the unprecedented completeness and taxonomic resolution in dietary descriptions from the DNA‐metabarcoding method was also critical for this purpose. This study has attempted to fill notable knowledge gaps in GSL trophic ecology, especially at broader spatial scales. We expect this knowledge to be further applied to the management and conservation not only of this endemic species, but also of other top‐predators living in fragile ecosystems.

## AUTHOR CONTRIBUTIONS

**Diego O. Urquía:** Conceptualization (equal); data curation (equal); formal analysis (equal); funding acquisition (equal); investigation (equal); methodology (equal); visualization (equal); writing – original draft (equal). **Sten Anslan:** Formal analysis (equal); investigation (equal); methodology (equal); writing – review and editing (equal). **Pacarina Asadobay:** Data curation (equal); investigation (equal); writing – review and editing (equal). **Andrés Moreira‐Mendieta:** Data curation (equal); writing – review and editing (equal). **Miguel Vences:** Conceptualization (equal); investigation (equal); methodology (equal); resources (equal); supervision (equal); writing – review and editing (equal). **Jaime A. Chaves:** Investigation (equal); writing – review and editing (equal). **Diego Páez‐Rosas:** Conceptualization (equal); formal analysis (equal); funding acquisition (equal); investigation (equal); project administration (equal); resources (equal); supervision (equal); writing – review and editing (equal).

## CONFLICT OF INTEREST STATEMENT

The authors declare no competing interests.

### OPEN RESEARCH BADGES

This article has earned an Open Data badge for making publicly available the digitally‐shareable data necessary to reproduce the reported results. The data is available at https://www.ncbi.nlm.nih.gov/bioproject/PRJNA947474/; https://www.ncbi.nlm.nih.gov/nuccore/OQ725637; https://www.ncbi.nlm.nih.gov/nuccore/OQ725638.

## Supporting information


Data S1–S2.

## Data Availability

Raw sequencing data is shared at the NCBI Sequence Read Archive (SRA; PRJNA947474; https://www.ncbi.nlm.nih.gov/bioproject/PRJNA947474/); 16S sequences generated for *P. clemensi* and *O. oculifer* are uploaded at the NCBI Genbank repository (OQ725637: https://www.ncbi.nlm.nih.gov/nuccore/OQ725637; OQ725638: https://www.ncbi.nlm.nih.gov/nuccore/OQ725638). Other data will be shared on reasonable request to the corresponding author.
